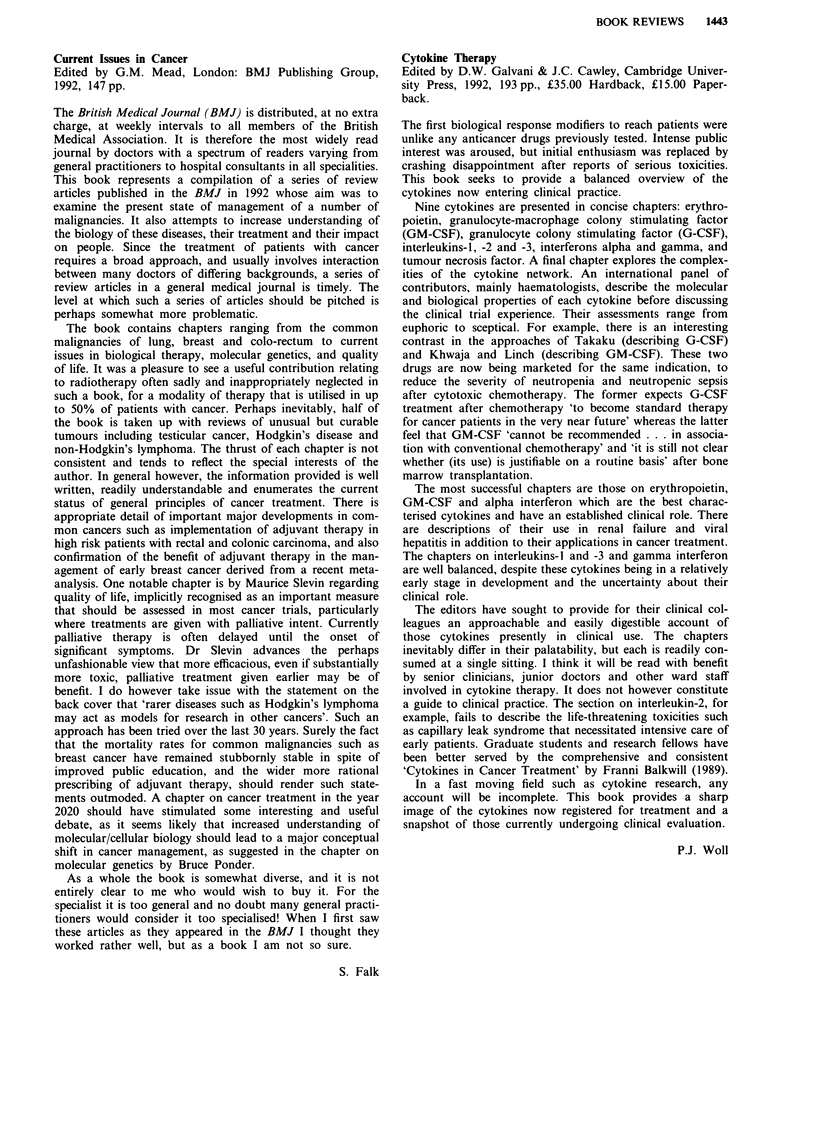# Cytokine Therapy

**Published:** 1993-06

**Authors:** P.J. Woll


**Cytokine Therapy**
Edited by D.W. Galvani & J.C. Cawley, Cambridge University Press, 1992, 193 pp., £35.00 Hardback, £15.00 Paperback.

The first biological response modifiers to reach patients were unlike any anticancer drugs previously tested. Intense public interest was aroused, but initial enthusiasm was replaced by crashing disappointment after reports of serious toxicities. This book seeks to provide a balanced overview of the cytokines now entering clinical practice.

Nine cytokines are presented in concise chapters: erythropoietin, granulocyte-macrophage colony stimulating factor (GM-CSF), granulocyte colony stimulating factor (G-CSF), interleukins-1, -2 and -3, interferons alpha and gamma, and tumour necrosis factor. A final chapter explores the complexities of the cytokine network. An international panel of contributors, mainly haematologists, describe the molecular and biological properties of each cytokine before discussing the clinical trial experience. Their assessments range from euphoric to sceptical. For example, there is an interesting contrast in the approaches of Takaku (describing G-CSF) and Khwaja and Linch (describing GM-CSF). These two drugs are now being marketed for the same indication, to reduce the severity of neutropenia and neutropenic sepsis after cytotoxic chemotherapy. The former expects G-CSF treatment after chemotherapy 'to become standard therapy for cancer patients in the very near future' whereas the latter feel that GM-CSF 'cannot be recommended . . . in association with conventional chemotherapy' and 'it is still not clear whether (its use) is justifiable on a routine basis' after bone marrow transplantation.

The most successful chapters are those on erythropoietin, GM-CSF and alpha interferon which are the best characterised cytokines and have an established clinical role. There are descriptions of their use in renal failure and viral hepatitis in addition to their applications in cancer treatment. The chapters on interleukins-1 and -3 and gamma interferon are well balanced, despite these cytokines being in a relatively early stage in development and the uncertainty about their clinical role.

The editors have sought to provide for their clinical colleagues an approachable and easily digestible account of those cytokines presently in clinical use. The chapters inevitably differ in their palatability, but each is readily consumed at a single sitting. I think it will be read with benefit by senior clinicians, junior doctors and other ward staff involved in cytokine therapy. It does not however constitute a guide to clinical practice. The section on interleukin-2, for example, fails to describe the life-threatening toxicities such as capillary leak syndrome that necessitated intensive care of early patients. Graduate students and research fellows have been better served by the comprehensive and consistent 'Cytokines in Cancer Treatment' by Franni Balkwill (1989).

In a fast moving field such as cytokine research, any account will be incomplete. This book provides a sharp image of the cytokines now registered for treatment and a snapshot of those currently undergoing clinical evaluation.

P.J. Woll